# Approximate Time to Steady-state Resting Energy Expenditure Using Indirect Calorimetry in Young, Healthy Adults

**DOI:** 10.3389/fnut.2016.00049

**Published:** 2016-11-03

**Authors:** Collin J. Popp, Jocelyn J. Tisch, Kenan E. Sakarcan, William C. Bridges, Elliot D. Jesch

**Affiliations:** ^1^Metabolic Laboratory, Department of Food, Nutrition and Packaging Sciences, College of Agriculture, Forestry and Life Sciences, Clemson University, Clemson, SC, USA; ^2^Department of Biological Sciences, College of Agriculture, Forestry and Life Sciences, Clemson University, Clemson, SC, USA; ^3^Department of Mathematical Sciences, College of Engineering and Science, Clemson University, Clemson, SC, USA

**Keywords:** indirect calorimetry, resting energy expenditure, steady state, methodology

## Abstract

Indirect calorimetry (IC) measurements to estimate resting energy expenditure (REE) necessitate a stable measurement period or steady state (SS). There is limited evidence when assessing the time to reach SS in young, healthy adults. The aims of this prospective study are to determine the approximate time to necessary reach SS using open-circuit IC and to establish the appropriate duration of SS needed to estimate REE. One hundred young, healthy participants (54 males and 46 females; age = 20.6 ± 2.1 years; body weight = 73.6 ± 16.3 kg; height 172.5 ± 9.3 cm; BMI = 24.5 ± 3.8 kg/m^2^) completed IC measurement for approximately 30 min while the volume of oxygen (VO_2_) and volume of carbon dioxide (VCO_2_) were collected. SS was defined by variations in the VO_2_ and VCO_2_ of ≤10% coefficient of variation (%CV) over a period of five consecutive minutes. The 30-min IC measurement was divided into six 5-min segments, such as S1, S2, S3, S4, S5, and S6. The results show that SS was achieved during S2 (%CV = 6.81 ± 3.2%), and the %CV continued to met the SS criteria for the duration of the IC measurement (S3 = 8.07 ± 4.4%, S4 = 7.93 ± 3.7%, S5 = 7.75 ± 4.1%, and S6 = 8.60 ± 4.6%). The current study found that in a population of young, healthy adults the duration of the IC measurement period could be a minimum of 10 min. The first 5-min segment was discarded, while SS occurred by the second 5-min segment.

## Introduction

Resting energy expenditure (REE) is the 24-h energy expenditure that reflects the total amount of energy needed to maintain basic physiological function and homeostasis (1, 2). REE is the largest component of total energy expenditure (TEE), and other components include the thermic effect of food and physical activity energy expenditure. REE is an important measure for clinicians and researchers who provide nutrition support in order to maintain energy balance and establish a physiological baseline of energy expenditure (3). The appropriate method for measuring REE in large observational trials is indirect calorimetry (IC). IC measures oxygen consumption (VO_2_) and carbon dioxide production (VCO_2_) and uses mathematically derived equations to estimate REE using a metabolic cart (1, 4). Traditionally, IC measurements require an individual lay supine in the postabsorptive state for 30–60 min while both VO_2_ and VCO_2_ are recorded (5–9).

In order for IC measurements to be valid and minimize variability, a stable measurement period or steady state (SS) should be achieved during the IC measurement (2, 5, 10). SS has been defined by achieving a ≤10% coefficient of variation (CV) for a specific amount of time using multiple variables, such as VO_2_, VCO_2_, and minute ventilation (VE). Horner et al. found that a greater percent of postmenopausal women met SS criteria of <10% variation during a 5-min period compared with a 10-min period after 30 and 45 min (11). McClave et al. found that the average REE for the SS period defined by a change in VO_2_ and VCO_2_ of <10% variation correlated best with the measured 24-h TEE (10). Therefore, a suitable definition for SS is a 5-min period with %CV <10% and is a useful time period to estimate REE (5, 12).

The total time period for IC measurement should be minimized in order to create a suitable testing environment for both the participant and investigator. In addition, standardizing the IC measurement protocol with the SS criteria will maximize compatibility and repeatability between researchers. Prior studies assessing the SS methodology of IC have been limited to non-healthy or critically ill patients, postmenopausal women, and older adults (>60 years) (5, 11–14). In an evidence analysis review, Fullmer et al. determined the duration of IC measurement to achieve SS in healthy and non-critically ill adults. They concluded that a 4-min SS period, after discarding the first 5 min of an IC measurement, is acceptable for estimating resting metabolic rate (2). These conclusions, however, were based on limited/weak evidence (grade III). In addition, only one study has examined the time required to reach SS in young, healthy adults (4).

The aims of this prospective study are to determine an approximate time to reach SS using open-circuit IC and establish the appropriate duration of SS needed to estimate REE. To achieve these aims, a 30-min IC measurement with no prior rest was conducted in young, apparently healthy adults. Based on the results of Borges et al., we hypothesized that the IC measurement length will be 10 min, while SS will be achieved during the second 5 min (4).

## Materials and Methods

### Participants

The participants were young, apparently healthy males and females who were recruited *via* flyers, electronic mail, and word-of-mouth as volunteers. Inclusion criteria included any undergraduate student between the ages of 18 and 30 years. Participants were excluded if they were pregnant, lactating, or were present with an acute or chronic disease (i.e., hyperthyroidism) that may affect REE. Two different undergraduate technicians (tech 1 and tech 2) collected data at two separate occasions. Tech 1 collected data from March 2014 to May 2014, and tech 2 collected data from November 2014 to October 2015. A total of 103 participants volunteered for this study, 55 males and 48 females. The Institutional Review Board at Clemson University approved all study procedures for human participants. No incentives were provided to participants, but participants received their REE results after completing the IC measurement. All participants signed a written informed consent document prior to testing.

### Indirect Calorimetry Measurement and Steady-state Definition

Prior to performing IC measurements, the technicians were trained to use the metabolic cart. They read the manufacturer’s instructions prior to performing multiple practice IC measurements on volunteers who were not included in the study. The same member of the research team trained both technicians, but the technicians were trained separately. After training, they were considered independent. Collin J. Popp or Elliot D. Jesch was present during all IC measurements.

The participants visited the metabolic laboratory between 07:00 a.m. and 11:00 a.m. after a 12-h fast and abstaining from any form of physical activity (other than walking) prior to completing a ventilated, open-circuit IC measurement. Participants were asked to remove excess clothing (e.g., jacket and shoes) and had their height (centimeters) and weight (kilograms) measured using a scale with stadiometer (SECA 763 digital scale, Chino, CA, USA). Then, participants were asked to lay supine on a padded table, and a rigid plastic canopy with a comfortable, flexible seal was placed over the head and torso. Expired gases were analyzed using a calibrated TrueOne^®^ 2400 metabolic cart (ParvoMedics TrueOne^®^, Murray, UT, USA). The gas analyzers, flow rate, and volume were calibrated as per manufacturer’s recommendations. To ensure quality control between participants and technicians, gas analyzers were calibrated to <0.1% standard gas and flow rate and volume were calibrated to ≤3% error. The ambient temperature was kept between 22 and 26°C, and humidity was maintained at roughly 60%. The participants were required to lay awake for approximately 30 min with no prior rest, and VE parameters were expressed as 30-s averages. REE (kilocalories per day) was calculated based on the Weir equation (15). The steady-state definition (denoted SS) was based on variations in the VO_2_ and VCO_2_ of ≤10% CV over a period of five consecutive minutes (5, 12). For analysis, the 30-min test was divided into six 5-min segments defined as S1, S2, S3, S4, S5, and S6.

### Statistical Analyses

Variables are presented as mean ± SD for each segment and technician. Data were collected separately for both technicians then combined for analysis. The %CV was also calculated for each segment. To compare means among segments, Fisher’s ANOVA *F*-test and Student’s *t*-test were used. To compare %CV among segments, Kruskal–Wallis test and Wilcoxon rank-sum test was used. The reason for using Kruskal–Wallis and Wilcoxon was that %CV had a skewed distribution, so that ANOVA and *t*-tests were not appropriate. A linear regression model was developed to show the relationship between the SS %CV and participant characteristics. All statistical comparisons were made at the alpha level of 0.05. All statistical calculations were performed using JMP^®^ Pro 10 (SAS Institute Inc., Cary, NC, USA).

## Results

### Participants

A total of 100 participants completed the REE test, 54 males and 46 females. Ethnicity was not part of the data collection protocol, but most participants were observed to be Caucasian. One male and one female were screened but did not complete the IC test. Their demographic characteristics were not used in the final analysis. One female participant from tech 2’s sample completed the IC test but reported thyroid problems. Her demographics and REE data were excluded from the final analysis. Table 1 gives the physical characteristics of all included participants. Detailed descriptions of the two technician’s demographics are provided in Table S1 in Supplementary Material.

**Table 1:** **Participant characteristics**.

	Mean ± SD
Age (years)	20.6 ± 2.1
Height (cm)	172.5 ± 9.3
Weight (kg)	73.6 ± 16.3
BMI (kg/m^2^)	24.5 ± 3.8
VO_2_ (ml/min)	249.0 ± 55.5
VO_2_ (ml/kg/min)	3.4 ± 0.6
VCO_2_ (ml/min)	221.3 ± 57.2
REE (kcal/min)	1.2 ± 0.3
REE (kcal/day)	1750.8 ± 395.7
REE (kcals/day/kg)	24.1 ± 4.2

*BMI, body mass index; VO_2_, volume of oxygen; VCO_2_, volume of carbon dioxide; REE, resting energy expenditure*.

### Steady State

**Figure 1:**
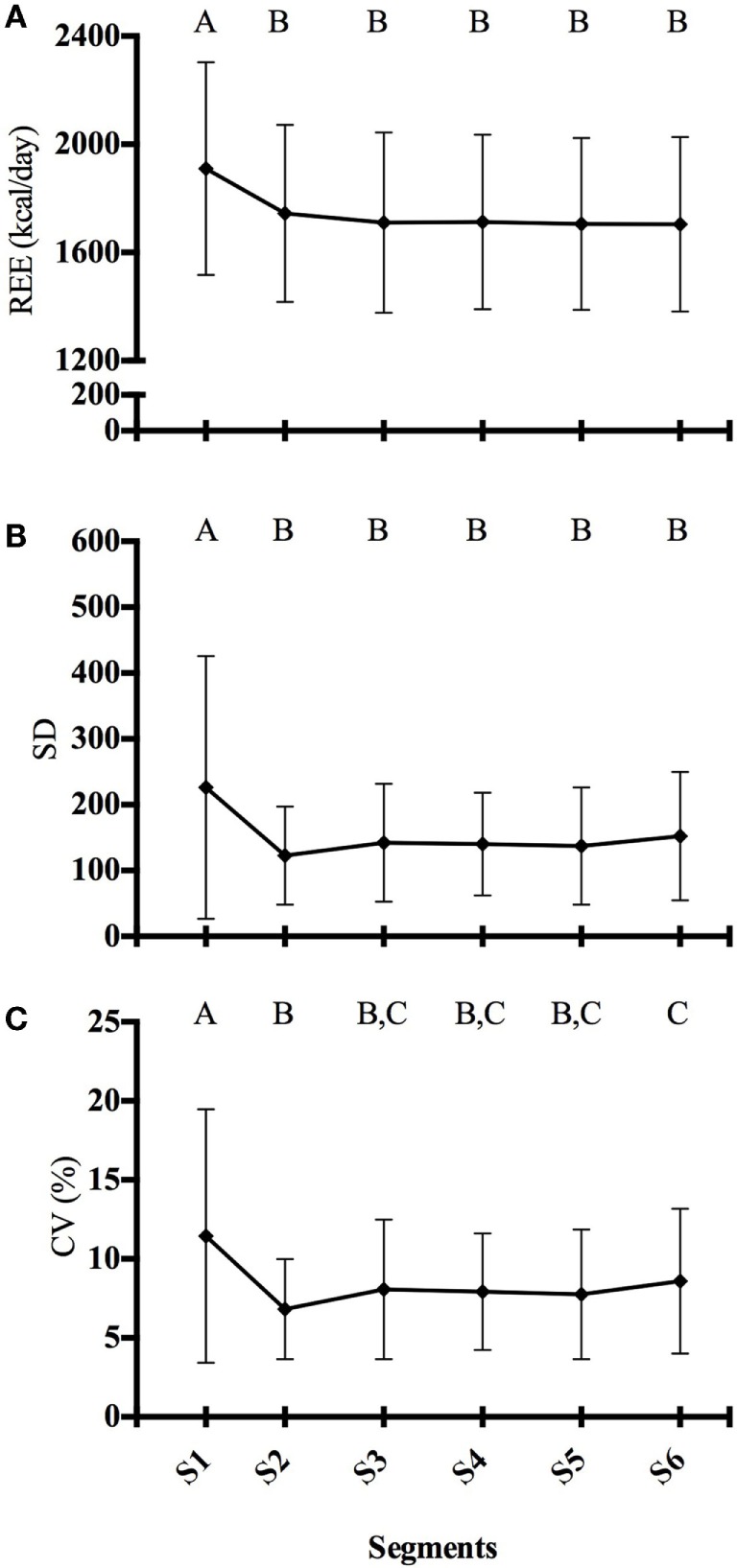
**Resting energy expenditure, SD, and percent coefficient of variation**. **(A)** REE, **(B)** SD, and **(C)** %CV. All variables are plotted over six 5-min segments (i.e., S1, S2, S3, etc.) Data represent mean ± SD. Means with different letters are statistical different (*P* < 0.05).

**Figure 2:**
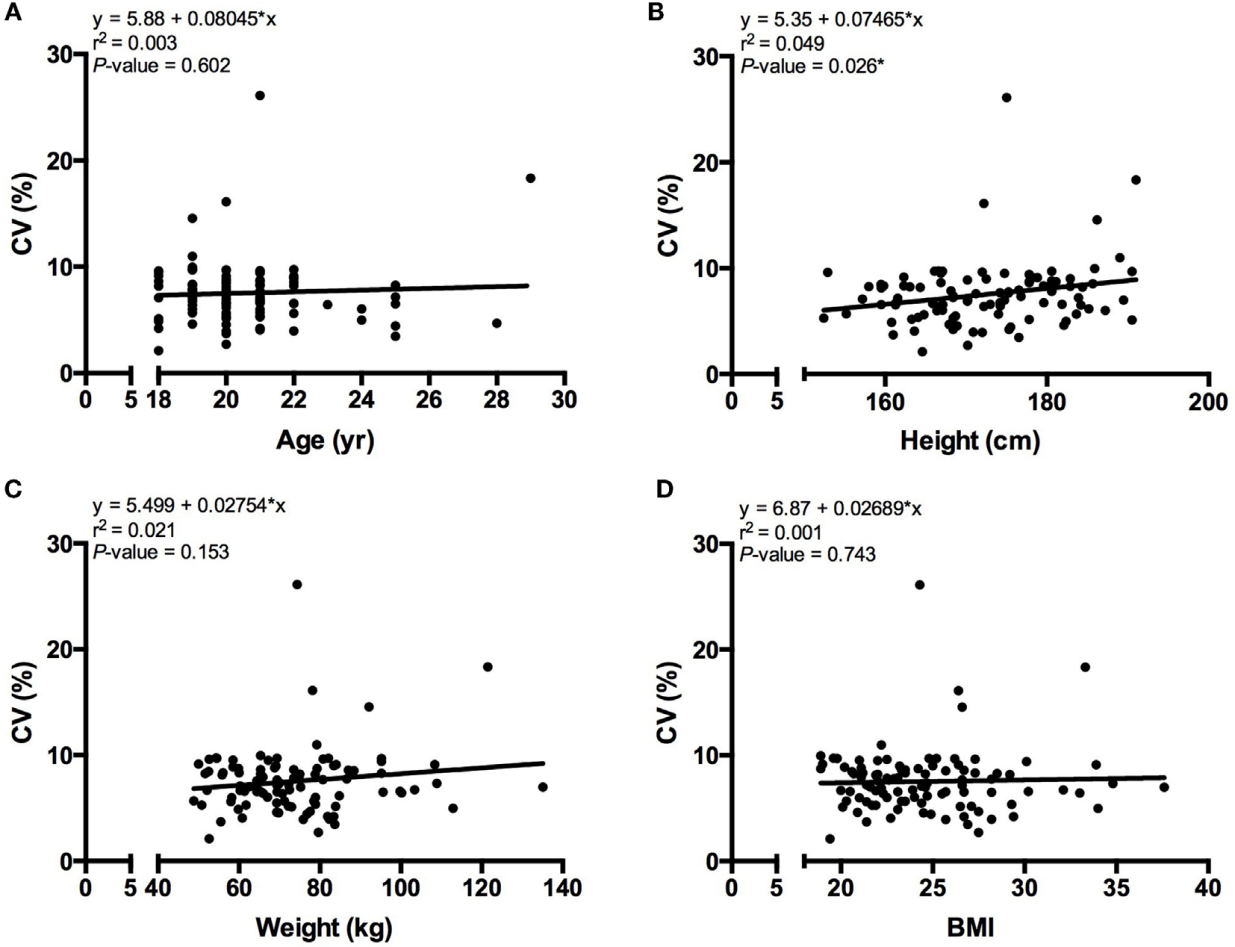
**Linear regression analysis**. A linear regression analysis comparing SS %CV to **(A)** age, **(B)** height, **(C)** weight, and **(D)** BMI (*P* < 0.05).

Figure 1 shows the changes in REE, SD, and %CV during the 30-min IC measurement. There was no difference in REE between segments S2, S3, S4, S5, and S6 (Figure 1A), which suggests that a 5-min measurement period is acceptable for estimating REE. This trend was similar with SD (Figure 1B). The first segment that met the SS criteria was S2 segment (Figure 1C). After the S2 segment, there is a slight increase in %CV as evidence by a significant difference between the S2 and S6 segments. However, both the S2 and S6 segments had a %CV ≤10. Figure 2 shows the linear regression between the SS %CV and participant characteristics. There was no significant relationship between participant characteristics and the SS %CV except for height.

Figure S1 in Supplementary Material shows changes in REE, SD, and %CV for each technician. The technicians achieved SS at different segments. Tech 1 achieved SS during the S1, and tech 2 achieved SS during S2 (Figures S1E,F in Supplementary Material). The effect size analysis shows a medium effect of technician (*d* = 0.40) on %CV during the S1 segment. The effect of technician on %CV was small during segments S2, S3, S4, and S6 (Table S1 in Supplementary Material).

## Discussion

The current prospective study aimed to determine the approximate time to reach SS using open-circuit IC and to establish the appropriate duration of SS needed to estimate REE. The results of the study support the hypothesis that a minimum IC measurement of 10 min, discarding the first 5-min, is acceptable for estimating REE. These results are supported by a non-significant change in mean REE after the S1 segment. In addition, the %CV for each 5-min segment after the first segment continued to meet the SS criteria. The linear regression analysis shows a statistically significant relationship only between participant height and SS %CV, whereas all other participant’s characteristics were non-significant. The participants of both technicians achieved SS at different segments. However, the recommendations we propose would ensure most participants for both tech 1 and tech 2 reach SS by the second 5-min segment.

We set to standardize the IC measurement protocol in the context of young, healthy adults. Prior studies were limited to non-healthy individuals, postmenopausal women, and older adults (5, 7, 8, 10, 11). For example, a 10-min IC measurement period, discarding the first 5 min, was acceptable for estimating resting metabolic rate in postmenopausal women (11). However, the protocol included a 30-min resting period before beginning the IC measurement.

Our results confirm these by Borges et al. found the best abbreviated time for an IC measurement is 10 min, with the first 5 min being discarded (4). Specifically, they measured REE in 37 Brazilian college students (27 males and 12 females) for 30 min. The %CV for the first 5-min was 19.9 ± 13.2% and was significantly greater than all other 5-min segments, while the second 5-min segment was the first abbreviated SS measurement period. Borges et al. was published during the writing of this manuscript.

The present study offers an approximate time to achieve SS. However, our study had a few limitations. We found that 5% of study participants did not reach SS within the 30-min testing period, and interestingly, all were males (3 from tech 1’s sample and 2 from tech 2’s sample). We also noticed that, in some cases, participants reached the SS criteria during the S1 or S2 segment, but their %CV subsequently increased above 10% CV as the measurement continued. This can be explained by the fact that participants were verbally notified during the IC measurement on the amount of time remaining. It is possible that this could have trigged anticipatory reaction leading to an increase in heart rate and increased fidgeting. However, these two variables were not measured. Two full-time undergraduate students (tech 1 and tech 2) completed the data collection procedure. The differences between tech 1 and tech 2 are presented in Supplementary Material. The undergraduate students were trained by the same individual, but at separate times. They may have lacked the necessary experience otherwise observed in an experienced graduate student or researcher. In addition, there was no study done to test for inter-tester reliability. Finally, the results also suggest minimal effect of light activity (e.g., walking, dressing) on REE as SS was achieved within the first 10-min, and participants did not spend the night in the metabolic laboratory. Future research should assess the correlation between the 10-min IC measurement period and 24-h TEE. In addition, our recommended IC protocol should be assessed in a population of working age adults and metabolically stable older adults.

In conclusion, we found that in a population of young, healthy adults the duration of the IC measurement period when measuring REE can be as little as 10 min, with the first 5-min being discarded. SS occurred by the second 5-min segment and continued to meet the SS criteria. Standardizing the IC measurement provides repeatability among researchers who want to best estimate REE, and thus extrapolated to 24-h TEE. Also, this information is important for researchers and practitioners working in a variety of settings for acutely and timely examining energy expenditure for better nutrition and physical activity intervention recommendations.

## Author Contributions

JT and KS were the two technicians who collected the data. CP, WB, and EJ had substantial contributions to the design, analysis, and interpretation. CP was responsible for drafting and revising the study, and all the authors were involved in approval of the final document submitted. CP and EJ are accountable for resolving questions relating to the accuracy and integrity of the work.

## Conflict of Interest Statement

The authors declare that the research was conducted in the absence of any commercial or financial relationships that could be construed as a potential conflict of interest.

## Abbreviations

%CV, percent coefficient of variation; BMI, body mass index; IC, indirect calorimetry; REE, resting energy expenditure; SS, steady state; TEE, total energy expenditure; VCO_2_, volume of carbon dioxide; VO_2_, volume of oxygen.

## References

[1] Matarese LE. Indirect calorimetry: technical aspects. J Am Diet Assoc (1997) 97(10 Suppl 2):S154–60. 10.1016/S0002-8223(97)00754-2 9336580

[2] Fullmer S Benson-Davies S Earthman CP Frankenfield DC Gradwell E Lee PSP Evidence analysis library review of best practices for performing indirect calorimetry in healthy and non-critically ill individuals. J Acad Nutr Diet (2015) 115(9):1417–46.e2. 10.1016/j.jand.2015.04.003 26038298

[3] Psota T Chen KY. Measuring energy expenditure in clinical populations: rewards and challenges. Eur J Clin Nutr (2013) 67(5):436–42. 10.1038/ejcn.2013.38 23443826 PMC3928639

[4] Borges JH Langer RD Cirolini VX Páscoa MA Guerra-Júnior G Gonçalves EM. Minimum time to achieve the steady state and optimum abbreviated period to estimate the resting energy expenditure by indirect calorimetry in healthy young adults. Nutr Clin Pract (2016) 31(3):349–54. 10.1177/0884533615627268 26888859

[5] Smyrnios NA Curley FJ Shaker KG. Accuracy of 30-minute indirect calorimetry studies in predicting 24-hour energy expenditure in mechanically ventilated, critically ill patients. J Parenter Enteral Nutr (1997) 21(3):168–74. 10.1177/0148607197021003168 9168370

[6] Schols AM Schoffelen PF Ceulemans H Wouters EF Saris WH. Measurement of resting energy expenditure in patients with chronic obstructive pulmonary disease in a clinical setting. J Parenter Enteral Nutr (1992) 16(4):364–8. 10.1177/0148607192016004364 1640635

[7] Leff ML Hill JO Yates AA Cotsonis GA Heymsfield SB. Resting metabolic rate: measurement reliability. J Parenter Enteral Nutr (1987) 11(4):354–9. 10.1177/0148607187011004354 3613036

[8] Fredrix EW Soeters PB von Meyenfeldt MF Saris WH. Measurement of resting energy expenditure in a clinical setting. Clin Nutr (1990) 9(6):299–304. 10.1016/0261-5614(90)90001-9 16837376

[9] Turley KR McBride PJ Wilmore JH. Resting metabolic rate measured after subjects spent the night at home vs at a clinic. Am J Clin Nutr (1993) 58(2):141–4. 8338039 10.1093/ajcn/58.2.141

[10] McClave SA Spain DA Skolnick JL Lowen CC Kieber MJ Wickerham PS Achievement of steady state optimizes results when performing indirect calorimetry. J Parenter Enteral Nutr (2003) 27(1):16–20. 10.1177/014860710302700116 12549593

[11] Horner NK Lampe JW Patterson RE Neuhouser ML Beresford SA Prentice RL. Indirect calorimetry protocol development for measuring resting metabolic rate as a component of total energy expenditure in free-living postmenopausal women. J Nutr (2001) 131(8):2215–8. 11481420 10.1093/jn/131.8.2215

[12] Frankenfield DC Sarson GY Blosser SA Cooney RN Smith JS. Validation of a 5-minute steady state indirect calorimetry protocol for resting energy expenditure in critically ill patients. J Am Coll Nutr (1996) 15(4):397–402. 10.1080/07315724.1996.10718615 8829096

[13] Reeves MM Davies PSW Bauer J Battistutta D Reducing the time period of steady state does not affect the accuracy of energy expenditure measurements by indirect calorimetry. J Appl Physiol (2004) 97(1):130–4. 10.1152/japplphysiol.01212.2003 15020579

[14] Petros S Engelmann L. Validity of an abbreviated indirect calorimetry protocol for measurement of resting energy expenditure in mechanically ventilated and spontaneously breathing critically ill patients. Intensive Care Med (2001) 27(7):1164–8. 10.1007/s001340100941 11534564

[15] de Weir JB New methods for calculating metabolic rate with special reference to protein metabolism. J Physiol (1949) 109(1–2):1–9. 10.1113/jphysiol.1949.sp004363 15394301 PMC1392602

