# Supplementary material for: Approximate Time to Steady-state Resting Energy Expenditure Using Indirect Calorimetry in Young, Healthy Adults
Source: Front Nutr. 2016 Nov 3;3:49. doi: 10.3389/fnut.2016.00049 (PMC5093115; doi:10.3389/fnut.2016.00049)
Supplement: Supplementary file 1 [file Table_1.DOC]

*Supplemental Material*

**Approximate time to steady state resting energy expenditure using indirect calorimetry in young, healthy adults.**

Collin J. Popp, Jocelyn J. Tisch, Kenan E. Sakarcan, William C. Bridges and Elliot D. Jesch^*^

Correspondence: Elliot D. Jesch [ejesch@clemson.edu](mailto:ejesch@g.clemson.edu)

Table 1: Participant demographics by technician

|  | Tech 1 (n=50) | Tech 2 (n=50) | *P*-value |
| --- | --- | --- | --- |
| Males | 23 | 31 | 0.109 |
| Females | 27 | 19 |
|  | Mean ± SD | Mean ± SD |  |
| Age (y) | 20.3 ± 1.5 | 20.9 ± 2.5 | 0.167 |
| Height (cm) | 171.2 ± 8.2 | 173.9 ± 9.9 | 0.133 |
| Weight (kg) | 70.0 ± 12.0 | 77.7 ± 18.7 | 0.016* |
| BMI (kg/m^2^) | 23.8 ± 2.9 | 25.4 ± 4.4 | 0.030* |
| VO_2_ (ml/min) | 243.5 ± 50.7 | 255.1 ± 59.3 | 0.207 |
| VO_2_ (ml/kg/min) | 3.522 ± 0.6 | 3.336 ± 0.6 | 0.024* |
| VCO_2_ (ml/min) | 217.2 ± 52.8 | 225.4 ± 61.1 | 0.352 |
| REE (kcal/min) | 1.188 ± 0.25 | 1.243 ± 0.3 | 0.229 |
| REE (kcal/day) | 1711.6 ± 360.8 | 1789.9 ± 424.0 | 0.229 |
| REE (kcals/day/kg) | 24.77 ± 4.0 | 23.40 ± 4.3 | 0.019* |

BMI, body mass index; VO_2_, volume of oxygen; VCO_2_, volume of carbon dioxide; REE, resting energy expenditure. **P* < 0.05
