# Supplementary material for: Approximate Time to Steady-state Resting Energy Expenditure Using Indirect Calorimetry in Young, Healthy Adults
Source: Front Nutr. 2016 Nov 3;3:49. doi: 10.3389/fnut.2016.00049 (PMC5093115; doi:10.3389/fnut.2016.00049)
Supplement: Supplementary file 2 [file Table_2.DOCX]

*Supplemental Material*

**Approximate time to steady state resting energy expenditure using indirect calorimetry in young, healthy adults.**

Collin J. Popp, Jocelyn J. Tisch, Kenan E. Sakarcan, William C. Bridges and Elliot D. Jesch^*^

Correspondence: Elliot D. Jesch [ejesch@clemson.edu](mailto:ejesch@g.clemson.edu)

Supplemental Table 2. Effect Size of %CV by technician

|  | %CV for REE Tech 1 | %CV for REE Tech 2 | Cohen’s *d* |
| --- | --- | --- | --- |
| Segment | Mean ± SD | Mean ± SD |  |
| S1 | 9.85 ± 4.0 | 13.0 ± 10.4 | 0.40 |
| S2 | 6.63 ± 4.8 | 7.00 ± 3.1 | 0.09 |
| S3 | 7.81 ± 4.3 | 8.34 ± 3.9 | 0.13 |
| S4 | 8.18 ± 4.8 | 7.68 ± 2.9 | 0.12 |
| S5 | 8.44 ± 4.6 | 7.07 ± 3.2 | 0.35 |
| S6 | 8.58 ± 3.3 | 8.61 ± 4.6 | 0.01 |

Effect size was calculated using Cohen’s *d* classification to compare technicians, where effect size of small (d = 0.2), medium (d = 0.5), large (d = 0.8) and very large (d > 1.3) are assigned. CV%, coefficient of variation
