# Supplementary figures and images for: Approximate Time to Steady-state Resting Energy Expenditure Using Indirect Calorimetry in Young, Healthy Adults
Source: Front Nutr. 2016 Nov 3;3:49. doi: 10.3389/fnut.2016.00049 (PMC5093115; doi:10.3389/fnut.2016.00049)

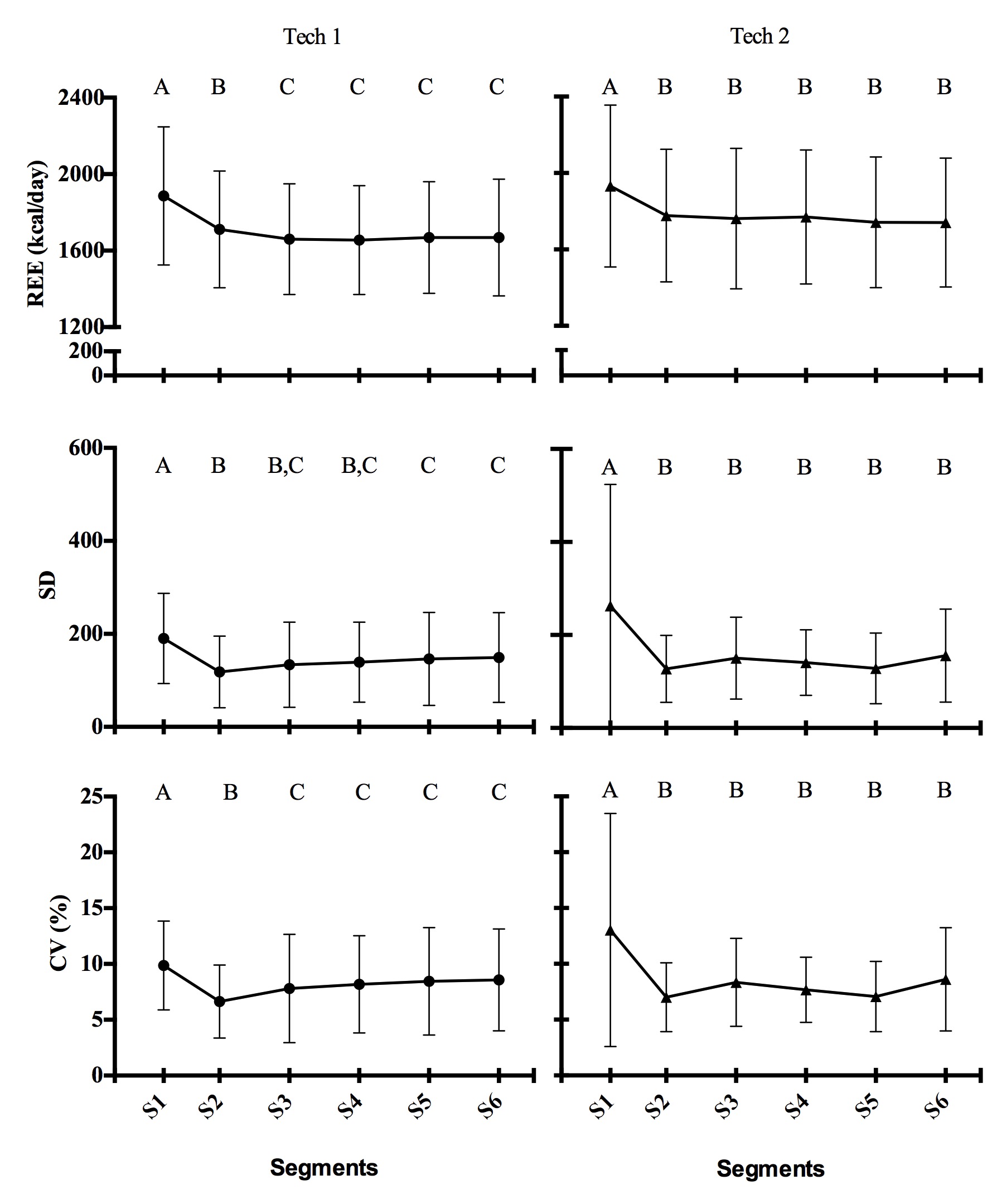

Supplement: Supplementary file 3 [file Image_1.JPEG]
